# Current Status of Proteomic Technologies for Discovering and Identifying Gingival Crevicular Fluid Biomarkers for Periodontal Disease

**DOI:** 10.3390/ijms20010086

**Published:** 2018-12-26

**Authors:** Sachio Tsuchida, Mamoru Satoh, Masaki Takiwaki, Fumio Nomura

**Affiliations:** Division of Clinical Mass Spectrometry, Chiba University Hospital, 1-8-1 Inohana, Chuo-ku, Chiba 260-8677, Japan; msatoh1995@yahoo.co.jp (M.S.); takiwaki@chiba-u.jp (M.T.); fnomura@faculty.chiba-u.jp (F.N.)

**Keywords:** periodontal disease, gingival crevicular fluid, proteomics, MALDI-TOF MS, LC-MS, MS, biomarker

## Abstract

Periodontal disease is caused by bacteria in dental biofilms. To eliminate the bacteria, immune system cells release substances that inflame and damage the gums, periodontal ligament, or alveolar bone, leading to swollen bleeding gums, which is a sign of gingivitis. Damage from periodontal disease can cause teeth to loosen also. Studies have demonstrated the proteomic approach to be a promising tool for the discovery and identification of biochemical markers of periodontal diseases. Recently, many studies have applied expression proteomics to identify proteins whose expression levels are altered by disease. As a fluid lying in close proximity to the periodontal tissue, the gingival crevicular fluid (GCF) is the principal target in the search for periodontal disease biomarkers because its protein composition may reflect the disease pathophysiology. Biochemical marker analysis of GCF is effective for objective diagnosis in the early and advanced stages of periodontal disease. Periodontal diseases are also promising targets for proteomics, and several groups, including ours, have applied proteomics in the search for GCF biomarkers of periodontal diseases. This search is of continuing interest in the field of experimental and clinical periodontal disease research. In this article, we summarize the current situation of proteomic technologies to discover and identify GCF biomarkers for periodontal diseases.

## 1. Introduction

**Figure 1:**
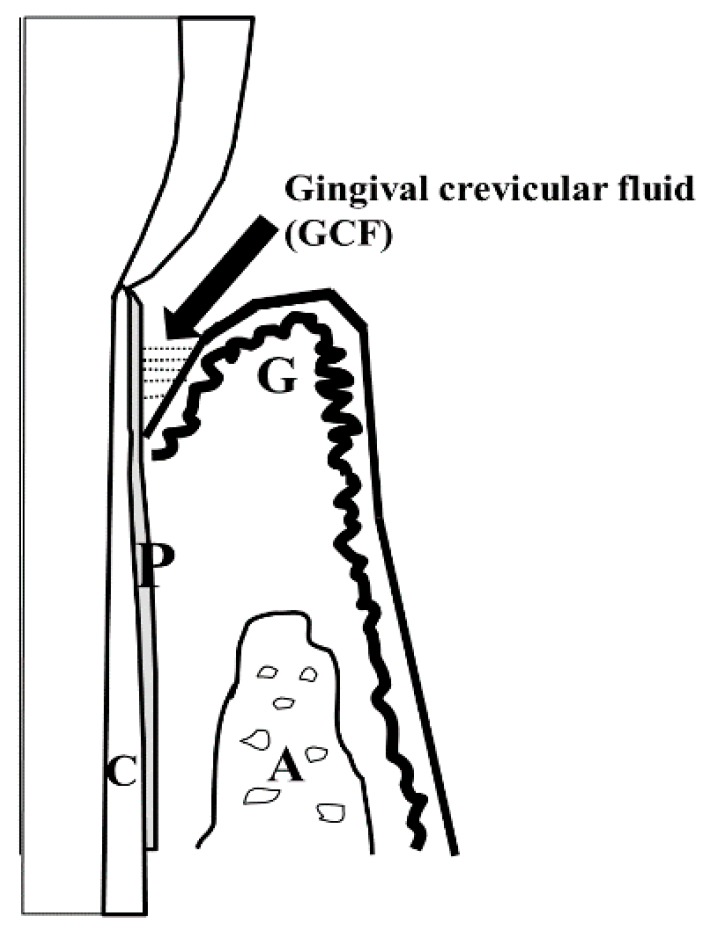
Schematic view of Gingival Crevicular Fluids (GCF). The GCF components may include breakdown products of host epithelial and connective tissues, factors produced by host cells in the periodontium, and products derived from subgingival microbial plaque. A, Alveolar bone; C, Cementum; G, Gingiva; P, Periodontal ligament.

Periodontal disease is characterized by the destruction of hard and soft connective tissue constituents of the periodontium [1,2,3,4,5,6,7,8,9]. Gingivitis always precedes periodontal disease and is characterized by gum inflammation. In contrast, periodontitis often refers to gum disease and the destruction of tissue and/or bone [10,11]. Bacterial pathogens are highly associated with the onset and progression of periodontal disease [12,13,14]. Various microbial dental plaque characteristics (e.g., development and heterogeneity; microbial succession, composition, and structure; and formation mechanisms) essentially precede the discussion of the microbial composition of the gingival crevice (subgingival plaque). Gingival crevicular fluid (GCF) is used to investigate proteomic changes that are associated with periodontal disease [15,16,17,18] (Figure 1). Small amounts of GCF are reportedly present in healthy gingiva, whereas large amounts are observed in inflamed regions [15,16,17,18]. This exudate constitutes various components, including leukocytes, proteases, cytokines, and prostaglandins [15,16,17,18]. Numerous factors, such as flow rate, gingival trauma, and repeat sampling, influence the volume of GCF [15,16,17,18].

Mass spectrometry (MS) is a powerful analytical tool used in numerous clinical laboratories worldwide. Recent advances in proteomics and MS technologies have provided promising methods for transdisciplinary discovery of novel markers.

A biomarker has to be useful in clinical practice and not only confirm a diagnosis that has already been established. A biomarker can be used instead of traditional diagnostic procedures when and where a periodontal professional is not available. A biomarker is meaningful in incipient clinical conditions and could be a predictive marker for disease progression. For instance, 20 years ago, GCF prostaglandins were found to have a predictive value for periodontitis progression.

A clear association has been reported between periodontal disease and other inflammatory conditions. Periodontal disease increases the risk of stroke, heart disease, diabetes, pulmonary problems, and other serious systemic conditions. Evidence suggests that proteomics facilitate the identification of GCF biochemical markers in patients with periodontal disease [19,20,21,22]. We focused on the present-day use of proteomic technologies for discovering and identifying GCF biomarkers for periodontal disease using matrix-assisted laser desorption/ionization time-of-flight mass spectrometry (MALDI-TOF MS) and liquid chromatography (LC) coupled with tandem mass spectrometry (MS/MS) (LC-MS/MS) in combination with proteomic technologies. In addition, we reviewed and discussed proteomic technologies that have been previously introduced to discover and identify prospective GCF biomarkers for periodontal diseases.

## 2. Human Gingival Crevicular Fluids (GCF) Samples

GCF may be considered as breakdown of the host epithelial and connective tissues, products of host cell in the periodontium, and products derived from the subgingival microbial plaque. A variety of proteins, which have been identified in GCF, play a critical role in periodontal tissue turnover. Consequently, the analysis of a biochemical marker in GCF may help in diagnosis as it also predicts the progression of periodontal disease [23]. Therefore, searches and investigations for potential diagnostic makers for periodontal diseases are warranted. The amount of GCF production is quite small and varies according to the size of the gingival sulcus. Few investigators have measured GCF; however, their observations have differed due to the variation in the GCF samples collected. Challacombe et al. reported that the mean GCF sample volume ranged from 0.43 to 1.56 μL in the proximal spaces from the molar teeth [24]. Moreover, Cimasoni et al. reported that they collected GCF from the sulcus of slightly inflamed gingiva and reported approximately 0.1 mg of GCF in 3 min [23], which is the amount of GCF affected by mechanical factors and habitats, such as smoke and circadian periodicity, and treatment (surgeries) of periodontal disease [22].

As a fluid lying in close proximity to periodontal tissue, GCF is the principal target in searches for biomarkers of periodontal diseases. There are many methods for collecting GCF, such as the use of paper strips, capillary tubes, paper cones, and gingival washing. Researchers have recently preferred to use paper strips [25]. GCF can be collected via suction, lavage, or absorption [26]. Suction methods usually employ microcapillaries/micropipettes. However, these methods are technique-sensitive and time consuming to ensure the accurate collection of a small amount of samples. In our laboratory, the absorption method is used because it is easy to perform, minimally invasive, and is traditionally the method of choice for GCF collection. Several different absorbent paper types are available and their qualities have been previously assessed [18]. Absorbent paper points have been used to collect subgingival plaque or other oral samples to analyze the presence of any microbes, particularly periodontal pathogenic bacteria [18,27]. Additionally, the extremely apical portions of the periodontal pocket are accessible, allowing sampling of this niche of front-line species. This procedure using paper points can minimize bleeding from the periodontal pocket during GCF sample collection and paper points visibly contaminated by blood are discarded. However, collecting GCF samples from periodontal pockets during the active phase of periodontitis without any bleeding is difficult.

GCF sample collection and preparation for MS methods have been evaluated. Preianò et al. [28,29] investigated the influence of collection methods and storage conditions on MALDI-TOF MS profiling of GCF. In terms of biobank study which storage condition is most appropriate remains to be clarified [30,31]. Sample collection and handling procedures can have a profound impact on the proteome [32]. To minimize possible protein degradation, samples should be frozen at the coldest possible temperature, particularly if they are to be stored for an extended period. Preianò et al. [28,29] assessed how storage conditions may influence the stability of the endogenous peptidome of GCF in gingivitis patients to define the storage conditions that avoid potential data misinterpretation. Mass spectra of five peptides were expressed differentially between gingivitis patients and healthy controls. Among these biomarkers, the C-terminal fragment of α-1-antitrypsin, namely C-36 peptide, and two different PTMs of the full-length S100A9 protein were found. Moreover, for all four peaks (*m*/*z* = 4328, 10,835, 12,689, and 13,153), a significant decrease in the peak area was observed for the storage time of 1 month at −20 °C. However, no significant changes were observed for storage at −80 °C after 1 month [28,29].

## 3. Discovery of Periodontal Disease Biomarkers by Proteomic Technology

### 3.1. Proteomic Technology (GC/MS, MALDI-TOF MS, and LC-MS/MS)

Proteomic analyzes detect biomarkers of numerous tumors and inflammatory diseases [33,34,35,36,37]. An alternative approach, termed shotgun proteomics, involves enzymatic digestion of whole proteins into small peptide fragments with uniform characteristics that are analyzed directly by LC-MS/MS [38,39]. Proteomic analysis, which includes MALDI-TOF/MS, gas chromatography MS (GC/MS), and LC/MS, has been applied increasingly to detect biomarker and diagnose diseases. Recently, these proteomic analyzes have become essential tools in oral sciences, such as periodontics [40,41], regardless of the level of specific proteome involved in GCF (Table 1).

To characterize the protein content of GCF in an unbiased and complete approach, Batschkus et al. [42] used MS, which allows not only identification but also quantification of these proteins. Therefore, a method to deplete serum albumin in GCF, and thus to enable detection of otherwise undetectable protein components in GCF by MS was necessary. Trichloroacetic acid/acetone precipitation was used successfully as a protein precipitation procedure for the efficient depletion of serum albumin from GCF samples. Moreover, Batschkus et al. [42] revealed that the precipitation step efficiently reduced the serum albumin content and increased subsequent protein identifications by 32%. They were able to identify 317 proteins using this technique. Furthermore, azurocidin (in periodontal research, there is increasing evidence that azurocidin serves as a potential biomarker for periodontitis [43]), Cathepsin B (Cathepsin B correlates positively with the clinical parameters of disease severity in untreated chronic periodontitis [44]), mitogen-activated protein kinases (mitogen-activated protein kinases play a significant role in the ontogeny of inflammation in periodontal disease resulting in alveolar bone loss [45]), coronin 1A (association of coronin 1A with greater pocket depths and signs of inflammation [46]), and MMP-9 (MMP-9 is involved in degradation of the PDL extracellular matrix during orthodontic tooth movement [47]) were identified in GCF from periodontally inflamed sites.

Ozeki et al. [48] reported on the efficacy of GC/MS that could be used to detect metabolites in GCF. GCF samples were collected from two diseased sites from each patient and from clinically healthy sites of volunteers. Overall, 19 metabolites were identified using GC/MS [48]. Total ion current chromatograms showed broad differences in the metabolite peak patterns between GCF samples obtained from healthy, moderate-, and deep-pocket sites. The intensity difference of some metabolites was significant at deep-pocket sites compared with that at healthy sites [48]. Metabolite intensities at moderate-pocket sites showed an intermediate profile between severely diseased and healthy sites [48]. The peak areas of putrescine, lysine, and phenylalanine were significantly higher in the deep-pocket site group compared with the healthy and moderate-pocket site groups [48]. The peak areas of other metabolites (ribose, taurine, 5-aminovaleric acid, and galactose) also were significantly higher in the deep-pocket site group compared with the healthy and moderate-pocket site groups. Ribose is a type of sugar normally produced in the body from glucose [49,50]. Ribose plays important roles in the synthesis of RNA, DNA, and the energy-containing substance adenosine triphosphate (ATP) [49,50]. D-Ribose is highly active in the glycation of proteins. Glycosylation has many different biological roles [51], varying from those related primarily to general effects of the size and shape of the glycan, such as protein folding and assembly of the protein complex, to those that depend upon the specific configuration of the branched glycan structures, such as cell recognition, cell–cell interaction, and immune response [52]. In the future, glycomics may be applied to the study of GCF from periodontal disease using MS.

Huynh et al. [53] compared the proteome composition of GCF obtained from healthy periodontal tissue and gingivitis and chronic periodontitis-affected sites. Each group consisted of 15 males. GCF was collected from each patient; pooled into healthy, gingivitis, and chronic periodontitis groups; and analyzed by gel electrophoresis and liquid chromatography electrospray ionization ion-trap tandem MS [53]. Cystatins B and S decreased in abundance from healthy to gingivitis groups and decreased even further in the chronic periodontitis group [53]. There was an increase in complement proteins from the healthy to gingivitis groups followed by a decrease in the chronic periodontitis group [53]. Immunoglobulins, keratin proteins, fibronectin, lactotransferrin precursor, 14-3-3 protein zeta/delta, neutrophil defensin 3, and α-actinin exhibited fluctuations in their expression levels among the three groups [53]. Sharma reported that the mean cystatin C concentration in GCF and serum was observed by enzyme-linked immunosorbent assay (ELISA) to be highest in the periodontitis group and lowest in the periodontally healthy group with intermediate concentration in the gingivitis and after periodontal therapy groups [54].

Yaprak et al. [55] evaluated protein profiles in gingiva obtained from periodontally healthy individuals who underwent a surgical crown lengthening procedure. Following 2-DE, in-gel tryptic digestion and MALDI-TOF/TOF steps were performed for protein identification. In total, 47 proteins were successfully identified and classified based on their classes, molecular functions, and involvement in biological processes and metabolic pathways [55]. Among them, 14-3-3 protein sigma, protein DJ-1, α-enolase, triosephosphate isomerase, superoxide dismutase, peroxiredoxin-1, protein S100-A9, galectin-7, annexin A2/A4, carbonic anhydrase 1, and chaperone proteins are worthy of attention [55].

Wellington et al. [56] selected eleven children (four boys, seven girls) with mixed dentition to participate in a split-mouth design study in which the deciduous second molar with radiographic evidence of root resorption served as the study site and the permanent first molar on the contralateral quadrant as the control site. GCF was collected using absorbing strips. In total, 22 samples were each analyzed by 1-dimensional LC-MS. Then, the remaining samples were pooled across the 11 patients and were analyzed by 2-dimensional LC-MS. Consequently, 2-dimensional LC-MS identified 2789 and 2421 proteins in the control and resorption pooled samples, respectively [56]. In addition, significantly up- and downregulated proteins were identified in teeth with root resorption [56]. Interestingly, many of these proteins are characteristically found in exosomes [56], which are small extracellular vesicles that modulate important functions in physiology and under pathological conditions of the central nervous system.

Carina et al. [46] hypothesized that the GCF proteome of healthy subjects qualitatively and quantitatively differs from the proteome of chronic periodontitis subjects. They used a quantitative proteomics approach based on label-free LC-MS on GCF of subjects with PH and chronic periodontitis [46]. In chronic periodontitis subjects, sites with clinical conditions differing from periodontal health, gingivitis, and periodontitis were assessed. Consequently, 230 proteins were identified, 145 in periodontal healthy cases, 214 in periodontitis, 154 in gingivitis, and 133 in healthy sites [46]. Four proteins were identified exclusively in periodontal healthy, forty-three in periodontitis, seven in gingivitis, and one in healthy cases. Four proteins were detected exclusively in the HH group, including keratin type II cytoskeletal 7, neuroblast differentiation-associated protein AHNAK, and two glial fibrillary acidic proteins. Compared with the healthy group, 35 and 6 proteins were more abundant in periodontitis and gingivitis (*p* < 0.001) groups, respectively; and 4, 15, and 37 proteins were less abundant in periodontitis, gingivitis, and healthy groups (*p* ≤ 0.01), respectively [46]. Proteins related to immune responses, such as Ig gamma-1 chain C region, Ig gamma-3 chain C region, lactoferroxin-C, lactrotransferrin, leukocyte elastase inhibitor, apolipoprotein E, alpha-1 antitrypsin, annexin, cathelicidin antimicrobial peptide, cathepsin G, coronin-1A, dermcidin isoform 2, heat shock protein beta-1, myeloperoxidase, neutrophil defensin 3, S100 A8, and S100 A9 were present in the samples obtained from deep pockets and/or had elevated relative abundance compared with samples from the healthy sites [46]. Moreover, myosin 9 and Annexin A1 showed significantly decreased relative abundance in P sites compared with the HH group [46].

### 3.2. Labeling Methods in Mass Spectrometry Based on Quantitative Proteomics (SILAC, iTRAQ, and TMT)

Proteins can be labeled metabolically with heavy or light isotope-containing growth media, and derivatization can occur following proteolytic digestion using isotopically distinct chemical labels or isobaric tags [57,58,59,60,61,62]. Other “label free” quantitation techniques eliminate labeling and instead rely on advanced software analyzes. These methods measure the relative concentrations of peptide analytes within two or more samples. Conversely, absolute quantitation techniques use internal standard peptides that have been prepared synthetically for selected reaction monitoring (SRM) or multiple reaction monitoring (MRM) analyzes and are increasingly becoming popular. Last, the MRM approach was combined with a quantitative MS technique involving stable isotope labeling by amino acids in cell culture (SILAC) [63,64,65]. Stable isotope labeling with SILAC experiments can quantify proteins and peptides accurately because samples are mixed early in the workflow; therefore, the variability contributed by sample preparation is minimized [63,64,65]. Moreover, SILAC increases spectral complexity because multiple isotopic clusters are created for each peptide, causing a redundancy in peptide identifications and reduced sampling depth.

Isobaric peptide labeling plays an important role in the relative quantitative comparisons of proteomic analysis. Isobaric labeling techniques use MS/MS spectra for relative quantification, which can be based on the relative intensities of reporter ions in the low mass region (e.g., iTRAQ and TMT) or on the relative intensities of quantification signatures throughout the spectrum owing to isobaric peptide termini labeling. Differentially labeled proteins do not differ in mass because of the isobaric mass design of iTRAQ reagents. Accordingly, their corresponding proteolytic peptides appear as single peaks in mass spectra. Owing to the fact that quantitative information is provided by isotope, encoded reporter ions that can only be observed in MS/MS spectra, Wiese et al. [66] analyzed the fragmentation behavior of ESI and MALDI ions of peptides generated from iTRAQ-labeled proteins using a TOF/TOF and/or a QTOF instrument.

A novel MS/MS-based analysis strategy using isotope labels, referred to as “tandem mass tags” (TMT), has been developed for the accurate quantification of peptides and proteins. A disadvantage of 2-DE analysis involves difficulties in analyzing proteins at low concentrations. The gel-free proteomic approach, which uses isobaric labeled reagents, such as TMT, may overcome this limitation [67,68,69,70]. The tags require novel methods of quantification analysis using tandem MS. The tags and analytical methods allow peptides from different samples to be identified by their relative abundance with greater ease and accuracy than other methods. The TMT expandable system allows for concurrent multiplexing of up to six different samples in a single experiment. Isobaric chemical tags are powerful tools that enable concurrent identification and quantitation of proteins in different samples by tandem MS. Dayon et al. [71] demonstrated the applicability of TMT to relatively quantify and identify biomarkers from biological fluids, such as cerebrospinal fluid. We also previously screened for novel biomarkers that could aid in the diagnosis of periodontal disease using the TMT method to compare proteins expressed in GCF samples from healthy controls with those expressed in GCF samples from patients with periodontal disease [72].

The introduction of isobaric tags for relative and absolute quantification (iTRAQ) of proteins in different samples was a major breakthrough in quantitative proteomics. The chemical tags attach to all peptides in a protein digest via free amines at the peptide N-terminus and on the side chain of lysine residues. Then, labeled samples are pooled and analyzed simultaneously. Moriya et al. [73] compared the protein profiles of GCF from deciduous (de-GCF) and permanent (pe-GCF) teeth in subjects with mixed dentition because the prevalence of periodontitis differs between children and adults. In this study, GCF sample pairs (de-GCF and pe-GCF) were taken from the same children and were compared by quantitative proteomics using the iTRAQ technique [73]. In total, 62 proteins were upregulated in de-GCF and 54 in pe-GCF. In particular, neutrophil-derived proteins, including myeloperoxidase and lactoferrin, were repeatedly higher in the de-GCF than in the pe-GCF teeth. These differences were verified using ELISA (*p* < 0.01). On the contrary, immunoglobulin components were upregulated in the pe-GCF [73].

Evaluation of quantitative changes of host and pathogenic bacteria-derived GCF proteins will advance our limited knowledge of the GCF protein composition and highlight specific alterations that occur in the disease state, which can be used as a biomarker discovery platform. Leandro et al. [74] investigated the quantitative proteome of GCF from 40 healthy individuals and 40 patients with periodontal disease using 320 GCF samples and stable isotope-labeling reagents, isotope-coded affinity tag, and mTRAQ with MS technology. They identified 238 distinct proteins, of which 180 were quantified in GCF of healthy and periodontal disease patients with an additional 26 and 32 distinct proteins found only in GCF of healthy or periodontal disease patients, respectively [74]. Forty-two pathogenic bacterial proteins and 11 yeast proteins were quantified. The data highlighted a series of proteins, such as host-derived Ig α-2 chain C, kallikrein-4, S100A9, transmembrane proteinase 13, peptidase S1 domain, several collagens, and pathogenic bacterial proteins (e.g., formamidase, leucine aminopeptidase, and virulence factor OMP85 from *P. gingivalis*), that were not quantified previously by large-scale MS approaches in GCF with relevance to periodontal disease, [74].

## 4. Conclusions

Compared to other biofluids, GCF protein profiles are yet to be explored. However, a rising number of studies have recently used expression proteomics to identify proteins whose abundance levels are altered by a disease. Proteomic analysis using 2-DE and MS monitor changes that occur in the protein component of tissues and subcellular compartments.

As noted in Table 1, proteomic technologies are used to discover and identify various prospective GCF biomarkers for periodontal diseases. These findings are a result of the evolution and improvements made in mass spectrometry devices and their applications. As quantitative proteomic technologies are rapidly advancing, an increasing number of novel periodontal disease markers are anticipated in the future. Current research suggests that proteins and peptides have beneficial effects on periodontal disease; however, further studies are needed. The diagnostic performance of GCF biomarker is evaluated via its sensitivity and specificity.

## Figures and Tables

**Table 1:** Application of proteomic technologies to discover and identify prospective biomarkers for periodontal diseases in gingival crevicular fluid.

Study	Methodology	Number of Identified Proteins	Candidate Protein Biomarkers of Periodontal Disease	Ref.
Carina et al., 2013	Gel-free: LC-MS/MS	230 proteins	Immune responses as Ig gamma-1 chain C region, Ig gamma-3 chain C region, Lactoferroxin-C, Lactrotransferrin, Leukocyte elastase inhibitor, Apolipoprotein E, Alpha-1 antitrypsin, Annexin, Cathelicidin antimicrobial peptide, Cathepsin G, Coronin-1A, Dermcidin isoform 2, Heat shock protein beta-1, Myeloperoxidase, Neutrophil defensin 3, S100 A8, S100 A9, Myosin 9, Annexin A1	[46]
Wellington et al., 2014	Gel-free: LC-MS/MS	2789 proteins	Exosomal proteins	[56]
Leandro et al., 2014	Gel-free: LC-MS/MS, stable isotope-labeling reagents, isotope-coded affinity tag, mTRAQ	238 proteins	Host-derived Ig α-2 chain C, Kallikrein-4, S100A9, Transmembrane proteinase 13, Peptidase S1 domain, Collagens, Pathogenic bacterial proteins	[74]
Huynh et al., 2014	1D-PAGE: LC-MS/MS	121 proteins	Cystatins B and S, Immunoglobulins, Keratin proteins, Fibronectin, Lactotransferrin precursor, 14-3-3 protein zeta/delta, Neutrophil defensin 3, α-actinin	[53]
Ozeki et al., 2016	Gel-free: GC/MS	19 metabolites	Putrescine, Lysine, Phenylalanine, Ribose, Taurine, 5-aminovaleric acid, Galactose	[48]
Moriya et al., 2017	Gel-free: LC-MS/MS, iTRAQ technique	62 proteins	Neutrophil-derived proteins, Myeloperoxidase, Lactoferrin,	[73]
Batschkus et al., 2018	1D-PAGE: LC-MS/MS	317 proteins	Azurocidin, Cathepsin B, Mitogen-activated protein kinases, Coronin 1A, MMP-9	[42]
Yaprak et al., 2018	2-DE; MALDI-TOF/TOF	47 proteins	14-3-3 protein sigma, Protein DJ-1, α-enolase, Triosephosphate isomerase, Superoxide dismutase, Peroxiredoxin-1, Protein S100-A9, Galectin-7, Annexin A2/A4, Carbonic anhydrase 1, Chaperone proteins	[55]

## Abbreviations

MS	Mass spectrometry
MALDI-TOF MS	Matrix-assisted laser desorption/ionization time-of-flight mass spectrometry
QTOF	Quadrupole TOF
ESI	Electrospray ionization
LC-MS/MS	Liquid chromatography (LC) coupled with tandem mass spectrometry (MS/MS)
SDS-PAGE	Sodium dodecyl sulfate polyacrylamide gel electrophoresis
2-DE	Two-dimensional electrophoresis
